# Artificial Intelligence in Cardiac Imaging With Statistical Atlases of Cardiac Anatomy

**DOI:** 10.3389/fcvm.2020.00102

**Published:** 2020-06-30

**Authors:** Kathleen Gilbert, Charlène Mauger, Alistair A. Young, Avan Suinesiaputra

**Affiliations:** ^1^Auckland Bioengineering Institute, University of Auckland, Auckland, New Zealand; ^2^Department of Anatomy and Medical Imaging, University of Auckland, Auckland, New Zealand; ^3^Department of Biomedical Engineering, King's College London, London, United Kingdom; ^4^Centre for Computational Imaging and Simulation Technologies in Biomedicine, School of Computing, University of Leeds, Leeds, United Kingdom; ^5^School of Medicine, Leeds Institute of Cardiovascular and Metabolic Medicine, University of Leeds, Leeds, United Kingdom

**Keywords:** cardiac anatomy, machine learning, left ventricle, MRI, statistical shape

## Abstract

In many cardiovascular pathologies, the shape and motion of the heart provide important clues to understanding the mechanisms of the disease and how it progresses over time. With the advent of large-scale cardiac data, statistical modeling of cardiac anatomy has become a powerful tool to provide automated, precise quantification of the status of patient-specific heart geometry with respect to reference populations. Powered by supervised or unsupervised machine learning algorithms, statistical cardiac shape analysis can be used to automatically identify and quantify the severity of heart diseases, to provide morphometric indices that are optimally associated with clinical factors, and to evaluate the likelihood of adverse outcomes. Recently, statistical cardiac atlases have been integrated with deep neural networks to enable anatomical consistency of cardiac segmentation, registration, and automated quality control. These combinations have already shown significant improvements in performance and avoid gross anatomical errors that could make the results unusable. This current trend is expected to grow in the near future. Here, we aim to provide a mini review highlighting recent advances in statistical atlasing of cardiac function in the context of artificial intelligence in cardiac imaging.

## Introduction

The main function of the heart is to pump blood to the lungs and body. In order to maintain the equilibrium state of normal blood circulation, the heart continuously adapts its structure, shape, and function in response to physiological challenges and long-term environmental factors. From the onset of injury or disease, the heart starts a cascade of structural and morphological adaptations, known as *cardiac remodeling*. Common cardiac remodeling includes left ventricular dilatation, increasing ventricular mass, hypertrophy, aortic dilation, and systolic/diastolic functional alterations. When this condition is prolonged, cardiac function may deteriorate until symptoms become clinically evident and may eventually lead to heart failure (1). Here, we define cardiac remodeling to encompass a wide spectrum of physiological processes from adaptive remodeling in athlete's hearts (2) and normal aging process (3) to adverse remodeling in hypertensive heart disorder (4) and ischemia (5). It is therefore critical in the management of patients with heart disease to identify and quantify the different types of cardiac remodeling and associations with environmental and clinical factors and to predict the likelihood of adverse outcomes in the future.

The associations between traditional risk factors of cardiovascular disease (including smoking, raised blood pressure, raised serum cholesterol, and diabetes mellitus) and developing cardiac disease were discovered from large epidemiological studies such as the Framingham Heart Study (6). To better understand the mechanism of subclinical disease, before symptoms are clinically evident, modern imaging examinations were later included, such as in the Multi-Ethnic Study of Atherosclerosis (MESA) (7) and the UK Biobank study (8). These large-scale studies have enabled a massive increase of imaging data available for the investigation of variations in cardiac geometry and function by using statistical shape analysis, as well as providing training data for machine learning algorithms.

Modern cardiac imaging modalities include echocardiography, computed tomography (CT), and magnetic resonance imaging (MRI). Each modality has its own advantages and disadvantages, but MRI has unique attributes over the other modalities that have enabled large-scale imaging studies in the general population, including the study of 6,000 preclinical subjects in the MESA and 100,000 asymptomatic subjects in the UK Biobank. MR images are acquired without ionizing radiation, and tomographic analysis can be performed without any geometrical assumption. In a single examination session, cardiac MRI can provide anatomical and functional images of the heart and great vessels in multiple views with high contrast-to-noise ratio, as well as high spatiotemporal resolution blood flow, microstructural tissue characterization, myocardial strain, blood perfusion, and scar images.

In this mini review, we focus on the rapid developments of machine learning combined with cardiac atlases. Although examples were taken mainly from cardiac MRI studies, these methods are generally extensible to other modalities. We first show how statistical shape analysis has enabled better understanding of cardiac shape remodeling within and between pathological groups. We then discuss current developments in machine learning to utilize the robustness of cardiac anatomy derived from statistical atlases to improve image analysis, including motion atlases to highlight the utility of dynamic data analysis vs. static analysis. Table 1 compares representative papers in each category. We conclude with a discussion of future perspectives of cardiac atlases in the context of artificial intelligence (AI) in cardiac imaging.

**Table 1:** Summary of cardiac atlas construction and deep learning methods with cardiac shape priors.

**Methods**	**Model**	**Strength**	**Weakness**	**Training cohorts**	**Availability**
**ATLAS CONSTRUCTIONS**					
Medrano-Gracia et al. (9)	LV	Mathematically defined cardiac shape model	Requires contours	1,991 MESA	CAP^a^
Mauger et al. (10)	LV, RV	Diffeomorphic	Requires segmentation	4,329 UK Biobank	CAP^a^
Bai et al. (11)	LV, RV	Volumetric model	Long breath-hold	1,093 healthy	ICL^b^
Hoogendoorn et al. (12)	LV, RV, LA, RA	High spatial resolution from CT	Small cohort; no healthy reference for CT	138 CAD	CISTIB^c^
**DEEP LEARNING IMAGE ANALYSIS WITH SHAPE PRIORS**					
Oktay et al. (13)	LV	Latent space regularization	Layers reduced for 3D	1,200 healthy	
Zotti et al. (14)	LV, RV	Simple adjustment of the U-Net	Single prior map	150 ACDC	VitaLabAI^d^
Chen et al. (15)	LV	Latent spaces for standard orientations	Each prior requires separate encoder; memory intensive	734 healthy	
Duan et al. (16)	LV, RV	2.5D	Computationally expensive; two stages of network (not end-to-end learning)	1,831 healthy; 649 pulmonary hypertension	Github^e^
**DEEP LEARNING SHAPE ANALYSIS**					
Attar et al. (17)	LV, RV	Direct prediction of the shape scores	Linear PCA shape model from non-linear deep learning network	3,500 healthy	
Clough et al. (18)	LV, RV	Interpretable	Latent reconstruction blurred	10,038 healthy; 778 CAD	
Painchaud et al. (19)	LV, RV	Augmented latent space	Requires three networks to train	150 ACDC	
**DYNAMIC ANALYSIS**					
Puyol-Antón et al. (20)	LV	Multimodalities	Separate pipelines; small cohort	50 healthy	
Bello et al. (21)	LV, RV	Survival loss on latent space	Displacement only	302 pulmonary hypertension	Github^f^
Peressutti et al. (22)	LV	Motion and clinical features	Small cohort	34 dyssynchrony	
Qin et al. (23)	LV	Joint motion and segmentation	2D + time	220 healthy	Github^g^

*LV, left ventricle; RV, right ventricle; LA, left atrium; RA, right atrium; CAD, coronary artery disease; MESA, multi-ethnic study of atherosclerosis; ACDC, automated cardiac diagnosis challenge*.

a *CAP, http://cardiacatlas.org*.

b *ICL,http://wp.doc.ic.ac.uk/wbai/data/*.

c *CISTIB, http://www.cistib.org/full-heart-pca-model-all-phases/en/full-heart-pca-model-all-phases*.

d *VitaLabAI, https://bitbucket.org/vitalab/vitalabai_public/src/master/VITALabAI/model/*.

e Github, https://github.com/j-duan/4Dsegment.

f Github, https://github.com/UK-Digital-Heart-Project/4Dsurvival.

g *Github, https://github.com/cq615/Joint-Learning-of-Motion-Estimation-and-Segmentation-for-Cardiac-MR-Image-Sequences*.

## Statistical Cardiac Atlases

Statistical atlases consist of maps of cardiac shape and function, which can be used to quantify the variation in the population and quantify the differences between cohorts. They can also be used to quantify shape scores in individual patients relative to standard population groups. For example, the Cardiac Atlas Project^1^ (24) provides repositories of thousands of cardiac MRI studies (25) and benchmark data for the development of automated analysis algorithms, including segmentation of images (26) and shape analysis (27).

**Figure 1:**
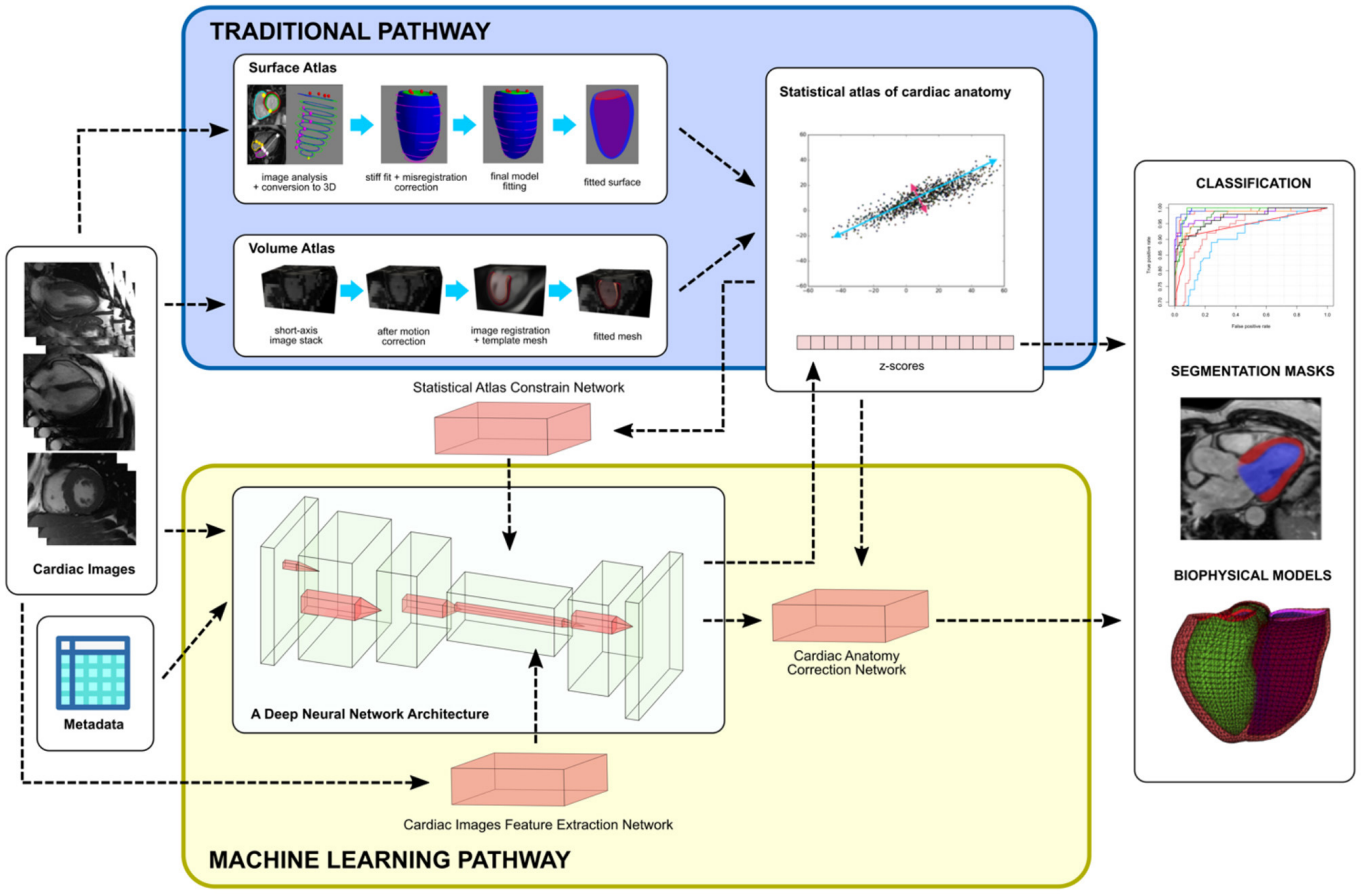
An overview of integrating statistical atlases of cardiac anatomy with deep neural network. Autoencoder is shown as the most common choice of deep learning architecture for latent space analysis. Although examples are shown with MR images, the methods are generally modality independent.

Two common atlas construction pipelines are shown in Figure 1, where both approaches lead to a comparable statistical analysis (28). In the first approach (9), images are analyzed to obtain the locations of cardiac landmarks (valve positions and the margins of the interventricular septum) and ventricular contours. The points are then mapped into 3D, and slice shifts due to breath-hold mis-registration are corrected. A 3D shape model template is then customized to the location of the landmarks and contours by minimizing the point-to-surface distances between the landmarks/contours and the model surfaces. Homologous points are then sampled from the surfaces and used to construct a point distribution model. This surface template fitting approach has also been translated to echocardiographic images where temporal resolution is much higher, as demonstrated in (20).

The second approach uses 3D images to establish a mean image template before generating cardiac mesh data. In (11), a high-resolution 3D MR template image and myocardial mesh are used. Each short axis image stack is then corrected for breath-hold mis-registration and registered to the template image using non-rigid image registration methods. For each case, a registration map is stored to give a mapping from subject space to template space at each voxel. The template mesh is then propagated to each subject using the inverse registration map. A point distribution model can then be calculated from the resulting homologous points. A similar approach was demonstrated in (12) by using CT images, with the advantage of high resolution and no breath-hold mis-registration in CT data.

Both these approaches benefit from recent advances in machine learning methods. Firstly, deep learning segmentation networks for cardiac images have been developed to enable fast generation of contours and landmarks (17, 29); and secondly, deep learning has enabled fast computation of registration maps, which can be trained without extensive manual image annotation using image similarity as the loss function (23, 30, 31).

## Atlas Measures of Cardiac Remodeling

Let *s* ∈ *R*^3*P*^ be a shape vector with *P* homologous points in 3D. To extract shape parameters from a cohort or pathology group, a linear generative model is commonly applied, that is,

(1) $$\begin{array}{l} s\approx \underline{s}+\Phi^{T}b \end{array}$$

where s ∈ *R*^3*P*^ is the mean shape estimated from the cohort, Φ ∈ *R*^*M* × 3*P*^ is the linear decomposition matrix (defining modes of shape variation), and *b* ∈ *R*^*M*^ is the shape parameter vector. If *N* is the number of patient shapes in the cohort and *M* < *N*, then Equation (1) is called a dimension reduction technique. Because each 3D point in this point distribution model encapsulates approximately the same anatomical location in the heart, the relative locations of neighboring positions are highly correlated, enabling the dimension reduction method to distill a small number of shape parameters.

The most common dimension reduction method is principal component analysis (PCA), whereby shape modes are ordered by the amount of variance explained. Most of the shape variations can then be explained in terms of the first few principal modes of variation. In the MESA baseline imaging study, the PCA mode explaining the most shape variation was associated with the size of the heart, even after correction for patient height (9). This is a common finding because the first mode often relates to the amplitude of the studied descriptors. The second mode was associated with sphericity. Clinically, these first two PCA modes are known to be associated with adverse outcomes in both symptomatic disease and asymptomatic cohorts (32–35).

PCA regression enables evaluation of the relationships between the PCA scores and clinical factors such as diabetes (9, 28). However, PCA is an unsupervised dimension reduction method, and component modes do not in general map to recognizable shape characteristics (9, 28). Supervised dimension reduction methods such as information maximizing component analysis have shown promise for quantifying the differences between a patient group and a control group, or two patient groups (36). Another approach is to combine dimensionality reduction with direct correlation with clinically defined remodeling indices such as ventricular volumes, wall thickness, and sphericity, by using the partial least squares method. Zhang et al. (37) applied this method in conjunction with a sequential orthogonalization algorithm to construct orthogonal shape scores, which are optimally matched with known clinical indices of remodeling. More general ways of characterizing the shape probability distribution have been investigated (38).

Gilbert et al. (28) found that both volume and surface cardiac atlases showed similar morphometric characteristics and similar relationships between risk factors and left ventricular shape. Thus, shape scores derived from atlases are robust to differences in construction methodology and quantify real anatomical relationships with cardiovascular risk factors. Morphometric scores were found to be more sensitive to cardiovascular risk factors than traditional measures of mass and volume. Mauger et al. (10) used a biventricular shape model to study right and left ventricular interactions in the UK Biobank study. A subdivision surface biventricular shape model was automatically customized to manually draw contours using a diffeomorphic least squares optimization algorithm. A control group sub-cohort consisting of 630 participants with no cardiovascular risk factors and normal cardiac parameters was used as a reference group to quantify shape differences due to traditional risk factors. Morphometric scores were computed using linear regression to quantify shape variations associated with prediction variables including sex, age, height, high cholesterol, high blood pressure, obesity, and smoking as well as diabetes, previous myocardial infarction, and angina. This regression approach enabled quantification of the effects of each prediction variable while controlling for the effects of the others.

In congenital heart disease, atlas-based analysis of shape variations can provide quantitative measures of deterioration before detection of symptoms. Sheehan et al. (39) developed a method for patient customization using a linear combination of database templates. This knowledge-based reconstruction method has shown accurate and rapid analysis of right ventricular shapes and volumes in patients with tetralogy of Fallot (39), dextro-transposition of the great arteries (40), and other types of congenital heart disease (41). A more dilated and spherical right ventricle was found in patients with transposition of the great arteries after atrial switch, with regional reduction in function at the base (42, 43). These methods assume that the patient heart geometry is accurately represented by a linear combination of cases in the database. An alternative approach is to jointly estimate the shape and the underlying statistical shape model so that the statistical model can be automatically updated while analyzing new cases (44). Shape model templates have been constructed to describe common congenital pathologies, such as congenitally corrected transposition of the great arteries, enabling a wide range of pathologies to be accurately characterized (45). In single-ventricle pathologies, with tricuspid atresia and Fontan repair, shape mode scores were able to quantify differences in shape and function, with more spherical ED shapes being associated with reduced longitudinal shortening (46). Atlas analysis in association with biomechanical analysis may be able to identify mechanisms underlying changes in function with developing disease (47).

## Deep Learning Networks With Cardiac Shape Priors

Deep learning is currently the state-of-the-art method for medical image feature extraction and supervised analysis. Its superior performance has surpassed any other traditional machine learning algorithms in many applications, including cardiac imaging (48, 49). This success is mainly attributed to the automatic generation of optimal features, rather than relying on handcrafted features. This means that without significantly modifying the architecture, deep learning allows transfer of techniques, thereby shifting the data domain from one application, for example, natural image analysis, to another, for example, cardiac imaging. In addition, transfer learning directly reuses a pretrained network and fine-tunes to a new application domain. Examples include transfer learning of retinal image segmentation into cardiac vessels (50) or predicting cardiovascular risk from retinal fundus images (51). This flexibility and reusability of deep neural network architectures have led to rapid development. However, there are some limitations. Deep learning is prone to overfitting and usually cannot infer the anatomical correctness of the prediction results. The network's parameters are also sensitive to the data or cohort used during training (implicit bias). Statistical atlases or shape priors can therefore be integrated with deep learning to overcome these limitations. Thus, anatomical correctness can be imposed by enabling the network to learn the biological constraints as well as the measurement correlations.

Machine learning methods can add new quantitative analysis techniques to examine the relationships between shape features and clinical status, in addition to the traditional methods of linear or logistic regression. These are now being applied to statistical shape atlases to characterize differences in patient groups and predict outcomes. In the STACOM 2015 shape analysis challenge (27), various machine learning algorithms were compared on a benchmark dataset, and 11 groups participated to determine cardiac shapes of patients with myocardial infarction from healthy subjects. Five groups used the *z*-scores (standardized *b* vector in Equation 1) in different ways to classify myocardial infarction shapes. The training accuracies ranged between 0.93 and 0.98, whereas the test accuracies were 0.83–0.98. Shape atlases have been useful in identifying genetic mutations affecting left ventricular (LV) mass (52). Shape features associated with disease can be interpreted through visualizations using deep generative networks (53).

Incorporating cardiac anatomy in deep learning was demonstrated by Oktay et al. (13) with an anatomically constrained neural network. Two separate autoencoder networks were appended after the final predicted segmentation mask and the ground truth mask layers, which extracted features from mask images separately. A global shape similarity loss function calculated from the output of autoencoder networks was introduced as a way to constrain the optimization to follow the same shapes as the ground truth. Their results showed improved super resolution and segmentation accuracies in the long-axis view^2^ by correcting mis-registration between image slices. Another shape-based loss function was also proposed by Yang et al. (54) to segment the right ventricle.

Alternatively, shape priors can be introduced directly inside a network (14–16). Zotti et al. (14) inserted a cardiac shape probability map before the final layer of a U-Net architecture to ensure that the output segmentation masks were valid. Chen et al. (15) also modified a U-Net architecture with cardiac shape priors, but they modified the bottom layer (feature extraction layer) by inserting short-axis and long-axis feature vectors trained independently from short-axis and long-axis cardiac MRI, respectively. Duan et al. (16) embedded a more specialized shape refinement subnetwork into the main segmentation and super resolution network. The subnetwork consisted of shape affine alignment, atlas selection, and non-rigid free form deformation registration operations. The network was able to generate smooth high-resolution 3D cardiac mesh data from low-resolution cardiac MRI.

## Deep Learning for Statistical Cardiac Atlases

The ability of deep learning to learn non-linear relationships between different data domains and the high focus on segmentation have enabled several studies to directly link cardiac imaging and statistical shape analysis. In Equation (1), patient-specific shape parameters with population reference of Φ are represented by *b* ∈ *R*^*M*^ vectors. A statistically plausible new shape of *s* can be generated by setting values of *b* within $\pm 2\sqrt{\sigma }$, where σ is the eigenvalues from the PCA. Shape generation can also be performed by sampling from a probability distribution function learned from an atlas (38).

Attar et al. (17) proposed a neural network model that learns how to directly predict shape parameters *b* given a combination of cardiac MRI and patient characteristics metadata [age, weight, height, body mass index (BMI), body surface area (BSA), heart rate, systolic blood pressure (SBP), diastolic blood pressure (DBP), sex, smoking status, and alcohol consumption]. Hence, the network was trained to predict statistically plausible *b* vector from images and metadata parameters to generate a 3D cardiac shape by using (Equation 1). Also, Clough et al. (18) used a variational autoencoder to generate interpretable representations of patients with low ejection fraction. This aids the interpretability of machine learning algorithm, which is vital to their acceptance in the clinical community.

A different approach to embed statistical shape parameterization into deep neural network was proposed by Painchaud et al. (19). A separate adversarial variational autoencoder was trained to generate a latent space of cardiac anatomy from mask images and was then connected to another anatomical variational autoencoder to correct errors after segmentation. Hence, this network (19) indirectly learned patient-specific parameters in the latent space without actually modeling how the latent space should be parameterized as in (17). The disentanglement of latent spaces is an active area of research and shows promise in factorizing anatomical representations from modality characteristics (55).

## Dynamic Atlases

Many of the features associated with cardiac pathology are manifest as changes in motion rather than changes in static shape. As the heart is responsible to deliver sufficient blood into the circulation system, the onset of cardiac diseases forces the heart to adapt its motion. Changes in cardiac shape deformation, myocardial strain, and strain rate are examples of important dynamic remodeling indices when building a cardiac motion or dynamic atlas. However, building a dynamic atlas is sometimes limited by the temporal resolution of the acquired imaging data, although combining two modalities, such as MRI and echocardiography (20), can increase the temporal resolution of the atlas considerably.

There are a significant number of cardiac applications that can get the benefit of machine learning from cardiac motion. In pulmonary hypertension, a motion atlas is combined with the latent space of autoencoder network to predict the survival rate (21). A machine learning system that combines motion atlas with non-motion data (ECG and clinical reports) has been demonstrated in the selection of patients with dyssynchrony for cardiac resynchronization therapy (22). The study of dynamic atlases will be a fruitful area of future research. Deep learning methods for combined shape and motion analysis are now being developed (23), which can be used to extend previous methods for motion atlasing (11).

## Discussion

A statistical atlas of cardiac anatomy is a powerful tool to analyze a patient-specific remodeling compared with the reference population. An abnormal cardiac shape can be quantified against a population reference, regional wall motion differences can be compared across pathological groups, and a hypothetical cardiac shape can further be predicted from a longitudinal study. Apart from that, a statistical atlas can be used as a reference by machine learning algorithms to constrain their analysis within valid anatomic boundaries.

In summary, we have reviewed three ways to integrate a statistical atlas into a machine learning framework. The first approach is to directly use individual shape atlas parameters, for example, the *z*-scores, as the training data. This approach needs homologous points generated from a shape modeling technique derived from images and a registration method to align points to remove variations in the global position and orientation. The effectiveness of this approach was demonstrated in the STACOM 2015 challenge. The second approach is to use statistical atlases as shape priors either as a way to measure shape similarity in a loss function or to add shape features to be learned inside the network. The third approach is to predict statistical shape parameters or a location in a shape-based feature space directly from images. This is a promising field for deep learning, because it can generate relationships between two completely different data domains.

In the future, statistical atlases show promise for augmenting deep learning methods, and vice versa. An atlas can add robustness to the prediction results because additional information on a reference population is included during the learning process. Atlases will also increase the interpretability of the AI process, which is critical for the acceptance of AI in health care.

## Author Contributions

All authors listed have made a substantial, direct and intellectual contribution to the work, and approved it for publication.

## Conflict of Interest

The authors declare that the research was conducted in the absence of any commercial or financial relationships that could be construed as a potential conflict of interest.

## Footnotes

^1^http://www.cardiacatlas.org

^2^In standard cardiac imaging acquisition, short-axis views show an image of the left and right ventricular chambers, and long-axis views typically show either two chambers (left ventricle and left atrium) or all four chambers in a single image. Short-axis views are generally perpendicular to the long-axis views.

## References

[1] Gjesdal O Bluemke DA Lima JA. Cardiac remodeling at the population level—risk factors, screening, and outcomes. Nat Rev Cardiol. (2011) 8:673–85. 10.1038/nrcardio.2011.154 22027657

[2] Maron BJ Pelliccia A. The heart of trained athletes: cardiac remodeling and the risks of sports, including sudden death. Circulation. (2006) 114:1633–44. 10.1161/CIRCULATIONAHA.106.613562 17030703

[3] Cohn JN Ferrari R Sharpe N. Cardiac remodeling—concepts and clinical implications: a consensus paper from an international forum on cardiac remodeling. J Am Coll Cardiol. (2000) 35:569–82. 10.1016/S0735-1097(99)00630-0 10716457

[4] Nwabuo CC Vasan RS. Pathophysiology of hypertensive heart disease: beyond left ventricular hypertrophy. Curr Hypertens Rep. (2020) 22:11. 10.1007/s11906-020-1017-9 32016791

[5] Sutton MG Sharpe N. Left ventricular remodeling after myocardial infarction: pathophysiology and therapy. Circulation. (2000) 101:2981–8. 10.1161/01.CIR.101.25.2981 10869273

[6] Kannel WB. Factors of risk in the development of coronary heart disease—six-year follow-up experience: the framingham study. Ann Intern Med. (1961) 55:33. 10.7326/0003-4819-55-1-33 13751193

[7] Bild DE. Multi-ethnic study of atherosclerosis: objectives and design. Am J Epidemiol. (2002) 156:871–81. 10.1093/aje/kwf113 12397006

[8] Petersen SE Matthews PM Bamberg F Bluemke DA Francis JM Friedrich MG. Imaging in population science: cardiovascular magnetic resonance in 100,000 participants of UK Biobank - rationale, challenges and approaches. J Cardiovasc Magn Reson. (2013) 15:46. 10.1186/1532-429X-15-46 23714095 PMC3668194

[9] Medrano-Gracia P Cowan BR Ambale-Venkatesh B Bluemke DA Eng J Finn JP. Left ventricular shape variation in asymptomatic populations: the multi-ethnic study of atherosclerosis. J Cardiovasc Magn Reson. (2014) 16:56. 10.1186/s12968-014-0056-2 25160814 PMC4145340

[10] Mauger C Gilbert K Lee AM Sanghvi MM Aung N Fung K. Right ventricular shape and function: cardiovascular magnetic resonance reference morphology and biventricular risk factor morphometrics in UK Biobank. J Cardiovasc Magn Reson. (2019) 21:41. 10.1186/s12968-019-0551-6 31315625 PMC6637624

[11] Bai W Shi W de Marvao A Dawes TJW O'Regan DP Cook SA. A bi-ventricular cardiac atlas built from 1000+ high resolution MR images of healthy subjects and an analysis of shape and motion. Med Image Anal. (2015) 26:133–45. 10.1016/j.media.2015.08.009 26387054

[12] Hoogendoorn C Duchateau N Sanchez-Quintana D Whitmarsh T Sukno FM De Craene M. A high-resolution atlas and statistical model of the human heart from multislice CT. IEEE Trans Med Imaging. (2013) 32:28–44. 10.1109/TMI.2012.2230015 23204277

[13] Oktay O Ferrante E Kamnitsas K Heinrich M Bai W Caballero J. Anatomically constrained neural networks (ACNNs): application to cardiac image enhancement and segmentation. IEEE Trans Med Imaging. (2018) 37:384–95. 10.1109/TMI.2017.2743464 28961105

[14] Zotti C Luo Z Lalande A Jodoin P-M. Convolutional neural network with shape prior applied to cardiac MRI segmentation. IEEE J Biomed Health Inform. (2019) 23:1119–28. 10.1109/JBHI.2018.2865450 30113903

[15] Chen C Biffi C Tarroni G Petersen S Bai W Rueckert D Learning shape priors for robust cardiac MR segmentation from multi-view images. In: Shen D Liu T Peters TM Staib LH Essert C Zhou S editors. Medical Image Computing Computer Assisted Intervention – MICCAI 2019. Cham: Springer International Publishing (2019). p. 523–31. 10.1007/978-3-030-32245-8_58

[16] Duan J Bello G Schlemper J Bai W Dawes TJW Biffi C. Automatic 3D Bi-ventricular segmentation of cardiac images by a shape-refined multi- task deep learning approach. IEEE Trans Med Imaging. (2019) 38:2151–64. 10.1109/TMI.2019.2894322 30676949 PMC6728160

[17] Attar R Pereañez M Bowles C Piechnik SK Neubauer S Petersen SE 3D Cardiac shape prediction with deep neural networks: simultaneous use of images patient metadata. In: Shen DT Liu TM Peters LH Staib C Essert S Zhou PT Yap A, editors. Medical Image Computing Computer Assisted Intervention – MICCAI 2019. Cham: Springer International Publishing (2019). p. 586–94. 10.1007/978-3-030-32245-8_65

[18] Clough JR Oksuz I Puyol-Antón E Ruijsink B King AP Schnabel JA Global local interpretability for cardiac MRI classification. In: Shen D Liu T Peters TM Staib LH Essert C Zhou S editors. Medical Image Computing Computer Assisted Intervention – MICCAI 2019. Cham: Springer International Publishing (2019). p. 656–64. 10.1007/978-3-030-32251-9_72

[19] Painchaud N Skandarani Y Judge T Bernard O Lalande A Jodoin P-M Cardiac MRI segmentation with strong anatomical guarantees. In: Shen D Liu T Peters TM Staib LH Essert C Zhou S editors. Medical Image Computing Computer Assisted Intervention – MICCAI 2019. Cham: Springer International Publishing (2019). p. 632–40. 10.1007/978-3-030-32245-8_70

[20] Puyol-Antón E Sinclair M Gerber B Amzulescu MS Langet H Craene MD. A multimodal spatiotemporal cardiac motion atlas from MR and ultrasound data. Med Image Anal. (2017) 40:96–110. 10.1016/j.media.2017.06.002 28646674

[21] Bello GA Dawes TJW Duan J Biffi C de Marvao A Howard LSGE. Deep-learning cardiac motion analysis for human survival prediction. Nat Mach Intell. (2019) 1:95–104. 10.1038/s42256-019-0019-2 30801055 PMC6382062

[22] Peressutti D Sinclair M Bai W Jackson T Ruijsink J Nordsletten D. A framework for combining a motion atlas with non-motion information to learn clinically useful biomarkers: application to cardiac resynchronisation therapy response prediction. Med Image Anal. (2017) 35:669–84. 10.1016/j.media.2016.10.002 27770718

[23] Qin C Bai W Schlemper J Petersen SE Piechnik SK Neubauer S Joint learning of motion estimation segmentation for cardiac MR image sequences. In: Frangi AF Schnabel JA Davatzikos C Alberola-López C Fichtinger G, editors. Medical Image Computing Computer Assisted Intervention – MICCAI 2018. Cham: Springer International Publishing (2018). p. 472–80. 10.1007/978-3-030-00934-2_53

[24] Fonseca CG Backhaus M Bluemke DA Britten RD Chung JD Cowan BR. The cardiac atlas project—an imaging database for computational modeling and statistical atlases of the heart. Bioinformatics. (2011) 27:2288–95. 10.1093/bioinformatics/btr360 21737439 PMC3150036

[25] Suinesiaputra A Medrano-Gracia P Cowan BR Young AA. Big heart data: advancing health informatics through data sharing in cardiovascular imaging. IEEE J Biomed Health Inform. (2015) 19:1283–90. 10.1109/JBHI.2014.2370952 25415993 PMC4659497

[26] Suinesiaputra A Cowan BR Al-Agamy AO Elattar MA Ayache N Fahmy AS. A collaborative resource to build consensus for automated left ventricular segmentation of cardiac MR images. Med Image Anal. (2014) 18:50–62. 10.1016/j.media.2013.09.001 24091241 PMC3840080

[27] Suinesiaputra A Dhooge J Duchateau N Ehrhardt J Frangi AF Gooya A. Statistical shape modeling of the left ventricle: myocardial infarct classification challenge. IEEE J Biomed Health Inform. (2018) 22:503–15. 10.1109/JBHI.2017.2652449 28103561 PMC5857476

[28] Gilbert K Bai W Mauger C Medrano-Gracia P Suinesiaputra A Lee AM. Independent left ventricular morphometric atlases show consistent relationships with cardiovascular risk factors: a UK biobank study. Sci Rep. (2019) 9:1130. 10.1038/s41598-018-37916-6 30718635 PMC6362245

[29] Bai W Sinclair M Tarroni G Oktay O Rajchl M Vaillant G. Automated cardiovascular magnetic resonance image analysis with fully convolutional networks. J Cardiovasc Magn Reson. (2018) 20:65. 10.1186/s12968-018-0471-x 30217194 PMC6138894

[30] Haskins G Kruger U Yan P Deep learning in medical image registration: a survey. Mach Vis Appl. (2020) 31:8 10.1007/s00138-020-01060-x

[31] de Vos BD Berendsen FF Viergever MA Sokooti H Staring M Išgum I. A deep learning framework for unsupervised affine and deformable image registration. Med Image Anal. (2019) 52:128–43. 10.1016/j.media.2018.11.010 30579222

[32] White HD Norris RM Brown MA Brandt PW Whitlock RM Wild CJ. Left ventricular end-systolic volume as the major determinant of survival after recovery from myocardial infarction. Circulation. (1987) 76:44–51. 10.1161/01.CIR.76.1.44 3594774

[33] Wong SP French JK Lydon A-M Manda SOM Gao W Ashton NG. Relation of left ventricular sphericity to 10-year survival after acute myocardial infarction. Am J Cardiol. (2004) 94:1270–75. 10.1016/j.amjcard.2004.07.110 15541243

[34] Bluemke DA Kronmal RA Lima JAC Liu K Olson J Burke GL. The relationship of left ventricular mass and geometry to incident cardiovascular events. J Am Coll Cardiol. (2008) 52:2148–55. 10.1016/j.jacc.2008.09.014 19095132 PMC2706368

[35] Ambale-Venkatesh B Yoneyama K Sharma RK Ohyama Y Wu CO Burke GL. Left ventricular shape predicts different types of cardiovascular events in the general population. Heart. (2017) 103:499–507. 10.1136/heartjnl-2016-310052 27694110

[36] Zhang X Ambale-Venkatesh B Bluemke DA Cowan BR Finn JP Kadish AH. Information maximizing component analysis of left ventricular remodeling due to myocardial infarction. J Transl Med. (2015) 13:343. 10.1186/s12967-015-0709-4 26531126 PMC4632345

[37] Zhang X Medrano-Gracia P Ambale-Venkatesh B Bluemke DA Cowan BR Finn JP. Orthogonal decomposition of left ventricular remodeling in myocardial infarction. GigaScience. (2017) 6:1–15. 10.1093/gigascience/gix005 28327972 PMC5791439

[38] Gooya A Lekadir K Castro-Mateos I Pozo JM Frangi AF. Mixture of probabilistic principal component analyzers for shapes from point sets. IEEE Trans Pattern Anal Mach Intell. (2018) 40:891–904. 10.1109/TPAMI.2017.2700276 28475045

[39] Sheehan FH Kilner PJ Sahn DJ Vick GW Stout KK Ge S. Accuracy of knowledge-based reconstruction for measurement of right ventricular volume and function in patients with tetralogy of fallot. Am J Cardiol. (2010) 105:993–9. 10.1016/j.amjcard.2009.11.032 20346319

[40] Morcos M Kilner PJ Sahn DJ Litt HI Valsangiacomo-Buechel ER Sheehan FH. Comparison of systemic right ventricular function in transposition of the great arteries after atrial switch and congenitally corrected transposition of the great arteries. Int J Cardiovasc Imaging. (2017) 33:1993–2001. 10.1007/s10554-017-1201-4 28668979

[41] Trzebiatowska-Krzynska A Driessen M Sieswerda GT Wallby L Swahn E Meijboom F. Knowledge-based 3D reconstruction of the right ventricle: comparison with cardiac magnetic resonance in adults with congenital heart disease. Echo Res Pract. (2015) 2:109–16. 10.1530/ERP-15-0029 26796613 PMC4677647

[42] Nyns ECA Dragulescu A Yoo S-J Grosse-Wortmann L. Evaluation of knowledge-based reconstruction for magnetic resonance volumetry of the right ventricle in tetralogy of fallot. Pediatr Radiol. (2014) 44:1532–40. 10.1007/s00247-014-3042-9 24986364

[43] Nyns ECA Dragulescu A Yoo S-J Grosse-Wortmann L. Evaluation of knowledge-based reconstruction for magnetic resonance volumetry of the right ventricle after arterial switch operation for dextro-transposition of the great arteries. Int J Cardiovasc Imaging. (2016) 32:1415–23. 10.1007/s10554-016-0921-1 27255743

[44] Stebbing RV Namburete AIL Upton R Leeson P Noble JA. Data-driven shape parameterization for segmentation of the right ventricle from 3D+t echocardiography. Med Image Anal. (2015) 21:29–39. 10.1016/j.media.2014.12.002 25577559

[45] Gilbert K Forsch N Hegde S Mauger C Omens JH Perry JC. Atlas-based computational analysis of heart shape and function in congenital heart disease. J Cardiovasc Transl Res. (2018) 11:123–32. 10.1007/s12265-017-9778-5 29294215 PMC5910190

[46] Farrar G Suinesiaputra A Gilbert K Perry JC Hegde S Marsden A. Atlas-based ventricular shape analysis for understanding congenital heart disease. Prog Pediatr Cardiol. (2016) 43:61–9. 10.1016/j.ppedcard.2016.07.010 28082823 PMC5222611

[47] Salehyar S Forsch N Gilbert K Young AA Perry CJ Hegde S A novel atlas-based strategy for understanding cardiac dysfunction in patients with congenital heart disease. Mol Cell Biomech. (2019) 16:179–83. 10.32604/mcb.2019.07384

[48] Bizopoulos P Koutsouris D. Deep learning in cardiology. IEEE Rev Biomed Eng. (2019) 12:168–93. 10.1109/RBME.2018.2885714 30530339

[49] Leiner T Rueckert D Suinesiaputra A Baeßler B Nezafat R Išgum I. Machine learning in cardiovascular magnetic resonance: basic concepts and applications. J Cardiovasc Magn Reson. (2019) 21:61. 10.1186/s12968-019-0575-y 31590664 PMC6778980

[50] Yu F Zhao J Gong Y Wang Z Li Y Yang F Annotation-free cardiac vessel segmentation via knowledge transfer from retinal images. In: Shen D Liu T Peters TM Staib LH Essert C Zhou S editors. Medical Image Computing Computer Assisted Intervention – MICCAI 2019. Cham: Springer International Publishing (2019). p. 714–22. 10.1007/978-3-030-32245-8_79

[51] Poplin R Varadarajan AV Blumer K Liu Y McConnell MV Corrado GS. Prediction of cardiovascular risk factors from retinal fundus photographs via deep learning. Nat Biomed Eng. (2018) 2:158–64. 10.1038/s41551-018-0195-0 31015713

[52] Biffi C de Marvao A Attard MI Dawes TJW Whiffin N Bai W. Three-dimensional cardiovascular imaging-genetics: a mass univariate framework. Bioinformatics. (2018) 34:97–103. 10.1093/bioinformatics/btx552 28968671 PMC5870605

[53] Biffi C Doumou G Duan J Prasad SK Cook SA O Regan DP. Explainable anatomical shape analysis through deep hierarchical generative models. IEEE Trans Med Imaging. (2020) 39:2088–99. 10.1109/TMI.2020.2964499 31944949 PMC7269693

[54] Yang H Liu Z Yang X Right ventricle segmentation in short-axis MRI using a shape constrained dense connected U-Net. In: Shen D Liu T Peters TM Staib LH Essert C Zhou S editors. Medical Image Computing Computer Assisted Intervention – MICCAI 2019. Cham: Springer International Publishing (2019). p. 532–40. 10.1007/978-3-030-32245-8_59

[55] Chartsias A Joyce T Papanastasiou G Semple S Williams M Newby DE. Disentangled representation learning in cardiac image analysis. Med Image Anal. (2019) 58:101535. 10.1016/j.media.2019.101535 31351230 PMC6815716

